# Antileishmanial Activity of *Lavandula angustifolia* and *Rosmarinus Officinalis* Essential Oils and Nano-emulsions on *Leishmania major* (MRHO/IR/75/ER)

**Published:** 2017

**Authors:** Azar SHOKRI, Majid SAEEDI, Mahdi FAKHAR, Katayoun MORTEZA-SEMNANI, Masoud KEIGHOBADI, Saeed HOSSEINI TESHNIZI, Hamid Reza KELIDARI, Siamak SADJADI

**Affiliations:** 1. Students Research Committee, School of Medicine, Mazandaran University of Medical Sciences, Sari, Iran; 2. Molecular and Cell Biology Research Center, Dept. of Parasitology, School of Medicine, Mazandaran University of Medical Sciences, Sari, Iran; 3. Dept. of Pharmaceutics, School of Pharmacy, Mazandaran University of Medical Sciences, Sari, Iran; 4. Dept. of Medicinal Chemistry, School of Pharmacy, Mazandaran University of Medical, Sciences, Sari, Iran; 5. Students Research Committee, School of Pharmacy, Mazandaran University of Medical Sciences, Sari, Iran; 6. Infectious and Tropical Diseases Research Center, Hormozgan University of Medical Sciences, Bandar Abbas, Iran

**Keywords:** *Leishmania major*, *Lavandula angustifolia*, *Rosmarinus officinalis*, Essential oil, Nano-emulsion

## Abstract

**Background:**

The aim of present study was to evaluate antileishmanial effects of *Lavandula angustifolia* (*L. angustifolia*) and *Rosmarinus officinalis* (*R. officinalis*) medicinal plants essential oils and nano-emulsions on *Leishmania major (L*. *major).*

**Methods:**

The present study was performed in Leishmaniasis Reference Lab at Mazandaran University of Medical Sciences, Iran during 2016–2017. The IC50 values were calculated in both the promastigote and amastigote stages in J774 macrophage in comparison with meglumine antimoniate (MA) as positive control. In addition, cytotoxicity effects of essential oils and nano-emulsions prepared from both plants against macrophages were evaluated.

**Results:**

Both essential oil and nano-emulsion of *Lavander* and *Rosemary* showed anti-leishmania activity on promastigote with IC_50_=0.11 μl/mL, IC_50_=0.26 μl/mL, and IC_50_=0.08 μl/mL respectively. Moreover, during amastigote assay, *Lavander* and *Rosemary* essential oils and nano-emulsion were effective at least in concentration of 0.12 μl/mL and 0.06 μl/mL (*P*=0.0001) respectively, on mean infected macrophages (MIR) and amastigotes in macrophages (*P*=0.0001). Additionally, cytotoxicity assay against macrophage revealed no toxicity on the host cells at IC_50_ concentrations.

**Conclusion:**

The nano-emulsions of both plants were more effective than essential oil in both MIR and amastigote. However, in comparison with MA, the *Lavander* essential oil is more effective in reducing MIR. *Rosemary* nano-emulsion reduced MIR significantly more than MA in concentration of 0.25 μl/mL (*P*<0.001). Further investigations are recommended to evaluate the effect of these medicinal plants in murine models.

## Introduction

Leishmaniases are neglected vector-borne parasitic diseases caused by an obligatory intracellular parasite of *Leishmania* spp. The infection is transmitted by bite of female sand fly during taking blood meals (1). The disease is endemic in 98 countries on five continents. More than 90% of cutaneous leishmaniasis (CL) forms appear in Afghanistan, Saudi Arabia, Algeria, Brazil, Iran, Iraq, Syria, and Sudan. In addition, the most cases of visceral form reporting from India, Sudan, and Brazil (1–3). Over the last decade, the incidence of CL has increased in more than half of the provinces (20 out of 32) in Iran (4).

The efficacy of current regimes including pentavalent antimony as first-line drugs has decreased and drug resistance has increased in many parts of the world. Second line drugs such as amphotericin B and pentamidine are very toxic (5).

The urgent need for substitute treatments has led to a program for screening natural products in leishmaniasis. Actually, the WHO recommended the use of traditional medicine in communities with poor health services (6). Some of herbal plants were used in traditional medicine for long times and today we know that they have anti-leishmanial effects as well and could be considered candidates for new drugs (7–9).

Rosemary is an herbal plant, commonly used for food flavoring and useful for the treatment of several diseases as an anti-inflammatory agent (10). It is useful in prevention or treatment of respiratory problems, peptic ulcers, tension headache, renal colic, heart disease and spasmogenic disorders (11).

Anti-parasitic activity of *Lavandula angustifolia* and *L. xintermedia* essential oils against three human protozoal pathogens *Giardia duodenalis*, *Trichomonas vaginalis* and the fish pathogen *Hexamita inflate* were evaluated (12). The aim of present study was to investigate anti-leishmanial effects of *Lavandula angustifolia* and *Rosmarinus officinalis* essential oils and nanoemulsions on *L. major*.

## Materials and Methods

### Preparation of nano-emulsion

The present study was performed in Leishmaniasis Reference Lab at Mazandaran University of Medical Sciences (MAZUMS), Iran during 2016–2017. The oil-in-water (o/w) nano-emulsions of *Lavander* and *Rosemary* essential oils were prepared using essential oil (1% w/w) and Span 60 (0.5% w/w) as oil phase, and mixture of Tween 80 (1% w/w) in deionized water as aqueous phase. The nanoemulsions were formulated as described previously (13). The physicochemical characterization was defined in terms of mean particle size, poly dispersity index and zeta potential using Zeta sizer Nano ZS (Malvern Instruments, UK). The results are the means of three determinations.

### Gas Chromatography and Mass Spectrometry

Gas chromatographic analysis was carried out on a Perkin-Elmer 8500 gas chromatograph with FID detector and a DB-5 capillary column (30 m × 0.25 mm; film thickness 0.25 μm).

Gas Chromatography-Mass Spectrometry (GC-MS) was performed on Hewlett Packard 6890 series, using a DB-5 capillary column (30 m × 0.25 mm, film thickness 0.25 μm) programmed as follows: 60 °C for 5 min and then up to 220 °C at 4 °C/min. The carrier gas was helium at a flow rate of 2 mL/min. The carrier gas was helium at a flow rate of 2 mL/min; split ratio, 1: 40; ionization energy, 70 eV; scan time, 1 sec; acquisition mass range, *m/z* 40–400.

Identification of Components:

The components of the oil were identified by their retention time, retention indices relative to C_9_-C_28_ n-alkanes, computer matching with the WILEY275.L library and as well as by comparison of their mass spectra with those of authentic samples or with data already available in the literature (14,15).

### Parasite culture

The Iranian strain of *L.major* (MRHO/IR/75/ER) was grown in RPMI-1640 medium (Gibco) with 20% heat-inactivated fetal bovine serum (Gibco), 100Upenicillin/mL, and 100mg streptomycin/*μ*L (Sigma) for preparation of sufficient promastigotes. Promastigotes were suspended in RPMI-1640 medium to adjust to a final concentration of 1 × 10^6^ parasites/mL.

### Promastigote assay

The in vitro evaluation of the anti-leishmanial activity of essential oils of the *Lavandula angustifolia* and *Rosmarinus officinal* and nano-emulsions of these plants, on parasites in the promastigote stage, were assessed using 96-well microplate. For the determination of the 50% inhibition concentration, each well was filled with 100 μL of the parasites suspension (1×10^6^ parasites/mL). Consequently, 10 μL of serial dilutions (from 1 to 0.0625 μl/mL) of selected plant essential oils and nano-emulsions were added to the same wells of microplate then the plate was incubated at 26 °C for 72 h. Wells without any component used as negative control and meglumine antimoniate (MA) (Glucantime®, Rhône–Poulenc, France) in well used as positive one.

### MTT assay

After 72 h of *Lavandula, Rosmary* and their nano components treatment, cell viabilities were assessed using the 3-(4,5-dimethylthiazol-2-yl)-2,5-diphenyltetrazolium bromide (MTT) micro method previously described (16,17). In addition, MA was used as a reference drug and MTT assay carried out consequently.

In brief, 10 ml of MTT (5 mg/mL) was added to each well and plates were further incubated for 4 h. The enzyme reaction was then stopped by addition of 100 μL of 50% isopropanol–10% sodium dodecyl sulfate. The plates were incubated for an additional 30 min under agitation at room temperature. Three replicates for each exposure concentration were examined. Absorbance values at 570 nm MTT (corrected at 630 nm) were measured using a microplate spectrophotometer (Bio-RAD Benchmark Plus). As a control, the activity of a drug alone in reagent was determined, and no substantial interaction was found. Then, The IC_50_ values of MA, *Lavandre* and *Rosemary* for promastigotes were calculated using the following formula:

X1(Log)−X2(Log)+((y1−y0/2)/(y1−y2)1)(X(Log50)=IC(Log))

### Amastigote assay

Macrophage line J774 A.1 (ATCC number TIB-67) was obtained from National Cell Bank of Iran (Pasteur Institute, Tehran, Iran). Macrophages were kept in RPMI-1640 medium. Cells were diluted in medium then following which viability test was performed by adding 90 μl of trypan blue solution (0.2%) in saline containing 0.01% sodium aside to 10 μL of cell suspension (10^6^ cells / mL). After 2 min, cells were counted under light microscope, where viability calculated as follows:

% Viability=(% of live cells/all counted cells ×100)

Briefly, 200 μL of the cells (10^6^ cells /mL) was added into 8chanel-chamber slides (SPL.Korea) and incubated at 37 °C with 5% CO_2_ for 2 h for cell adhesion. Promastigotes (10^7^/mL) were added to macrophages and incubated at 37 °C with 5% CO_2_ for 24 h. Then essential oils and nano-emulsions in several concentrations (10 μL) in medium added to the slides and incubated at 37 °C for 72 h. In addition, MA as a reference drug was added.

Dried slides were fixed with ethanol, stained with Wright-Giemsa and studied under light microscope. Macrophages containing amastigotes with no drugs and macrophages alone were considered as positive and negative controls, respectively. Drug activity was evaluated by counting the number of amastigotes in the macrophages by examining 100 macrophages and the number of macrophages was infected in 100 macrophages (MIR) (17).

### Cytotoxicity assay

In vitro toxicity against J774.A.1 macrophages was assessed with cells plated in 96-well plates at 2 × 10^5^ cells / well. After cell adherence, the medium was removed and replaced by the media containing IC_50_ concentration of each compound. The plates were incubated for 24 h at 37 °C in an incubator with 5% CO_2_. Control cells were incubated with culture medium plus DMSO. Cell viability was determined using MTT colorimetric assay.

### Data analysis

Indicator absorbance values at 72 h time points for MTT were analyzed using calculated using CalcuSyn version 2 software (Biosoft, UK). In addition, the SPSS software was used to analyze the data. ANOVA test, multiple comparison test, and t-test were used and *P*<0.05 was considered as a significant difference.

## Results

### Promastigote activity

The nano-emulsions were stable for 6 months at 4 °C and 25 °C. The particle sizes of *Lavander* and *Rosemary* essential oil, nanoemulsion were 104.2 ± 5.3 nm and 98.7 ± 6.4 nm respectively. The polydispersity index was 0.312± 0.09 and 0.298 ± 0.085 for *Lavander* and *Rosemary* nano particles. Zeta potential of these nano-emulsions was −15.8±1.2 mv and −17.3±1.4 mv respectively. The major compounds of the *Lavander* essential oil were 1.8-cineol (22.29%), Linalol (11.22%), camphor (7.88%), β-pinene (5.78%), α-terpineol (4.85%), α-pinene (4.56%), terpine-4-ol (4.19%) and borneol (4.03%). The major constituents of the essential oil were 1.8-cineol (15.96%), α-pinene (13.38%), camphor (7.87%), bornyl acetate (6.54%), verbenone (5.82%), borneol (5.23%), camphene (4.96%), and (E)-caryophyllene (3.8%).

The in vitro anti-leishmanial activities of the nano-emulsions of *Lavander* and *Rosemary* as well as essential oils were investigated against standard strain of *L. major*. For *Lavander* essential oil and nano-emulsion, the effective concentration with IC_50_=0.11 μl/mL achieved. For *Rosemary* essential oil, the effective concentration with IC_50_=0.26 μl/mL and its nano-emulsion IC_50_=0.08 μl/mL were achieved. The IC_50_ value of MA was 197 mg/ mL, is significantly higher than those of *Lavander* and *Rosemary* nano-emulsions and essential oils (*P*< 0.0001).

### Amastigote activity

The effect of essential oil and nanoemulsion of both *Lavander* and *Rosemary* on amastigote stage of parasite was evaluated by the mean infection rate of macrophages (MIR) and by the mean number of amastigotes per macrophage. Comparison of the MIR showed that different concentrations of *Lavander* and *Rosemary* essential oils significantly inhibited infection of macrophages by at least 0.12 μl/ Ml (*P*<0.0001) (Table 1). For nano-emulsion the effective concentration which significantly inhibited infection of macrophages were at least 0.06 μl/mL when compared with positive control (*P*<0.001). Moreover, comparing different concentrations of *Lavander* and *Rosemary* essential oils on the mean number of amastigotes in each macrophage was 0.12 μl/ mL as for nano-emulsion the mean number of amastigotes in each macrophage decreased at the concentration of 0.06 μl/ mL when compared with positive control (*P*<0.001) (Table 2). All results were compared with MA, which was the drug of choice in our study. The difference between *Lavander* essential oil and MA in reducing mean infection rate of macrophages (MIR) was significant in doses 1 μl/ mL (*P*=0.001) and 05 μl/ mL (*P*=0.044).

**Table 1:** Several concentrations and percent of infected macrophages (MIR) compared with control and MA

***Concentration (μl/mL)***	***Lavander Essential Oil MIR Mean± SD***	***Rosemary Essential Oil MIR Mean± SD***	***Lavander Nano-emulsion MIR Mean± SD***	***Rosemary Nano-emulsion MIR Mean± SD***
1	19.33*± 3.05	13.33*±1.52	ND	ND
0.5	24.33*±3.05	23.33*±3.05	ND	ND
0.25	32.00*±1.00	29.66*±2.08	37.33*±3.05	13.33*±1.53
0.125	44.33*±2.01	39.33*±6.02	46.33*±1.52	43.00*±6.93
0.0625	ND	ND	54.33*±2.08	54.33*±7.02
Control	85.70±2.08	85.70±2.08	85.70±2.08	85.70±2.08
MA(μg/mL)	30.33*±2.90	30.33*±2.90	30.33*±2.90	30.33*±2.90

Control= Infected macrophages without drug // ND= Not determined //MA= Meglumine antimoniate) Glucantime), *= *P*<0.05

**Table 2:** Several concentrations and number of amastigotes in macrophages compared with control and MA

***Concentration (μl/mL)***	***Lavander Essential Oil Amastigotes Mean± SD***	***Rosemary Essential Oil Amastigotes Mean± SD***	***Lavander Nano-emulsion amastigotes Mean± SD***	***Rosemary Nano-mulsion amastigotes Mean± SD***
1	1.70*±0 .10	1.34*±0. 08	ND	ND
0.5	2.01*±0 .07	1.67*±0. 15	ND	ND
0.25	2.41*±0. 18	2.06*±0. 03	1.73*±0.06	1.62*±0.04
0.125	2.69*±0.25	2.45*±0. 04	2.45*±0.05	1.79*±0.07
0.0625	ND	ND	3.02 ±0.07	2.07*±0.09
Control	3.70±.63	3.70±.63	3.70±.63	3.70±.63
MA (μg/mL)	1.70*±0.20	1.70*±0.20	1.70*±0.20	1.70*±0.20

Control= amastigotes in macrophages without drug // ND= Not determined //MA= Meglumine antimoniate) Glucan-time), *=*P*<0*.*05

In these concentrations, MIR was declined to 19.33% and 24.33% respectively which was lower than MA with 30.33%. However, in terms of the difference between *Rosemary* essential oil and MA in reducing MIR, same result achieved in reduced MIR. *Rosemary* essential oil reduced MIR to 13.33 % and 23.33, which was lower than MA with 30.33%. (*P*=0.0001). *Rosemary* essential oil in concentration of 1 μl/mL significantly decreased MIR more than *Lavander* (*P*=0.0001). *Lavander* and *Rosemary* nano-emulsions significantly reduced MIR and the number of amastigotes in macrophages in at least 0.06 μl/mL in compare with control (*P*=0.001). The difference between several concentrations of *Rosemary* nano-emulsion in concentration of 0.25 μl/mL in reducing amastigotes in macrophages and MA was significant (*P*=0.04). Moreover, *Rosemary* essential oil in concentration of 1μl/mL decreased amastigotes in macrophages significantly more than MA (*P*=0.04). *Rosemary* nano-emulsion reduced *amastigotes* in macrophages in concentrations of 0.25.0.125 and 0.0625 μl/mL significantly more than *Lavander* nano-emulsion (*P*=0.39) (Table 2).

## Discussion

Pentavalent antimonials as first-line drugs had been widely used for the treatment of CL all around the world for decades, but treatment failure occurs in endemic regions (18). Alternative drugs such as miltefosine, amphotericin B, and some azoles are used as second-line drugs for the treatment of disease (19). All of these drugs have serious side effects and are expensive and unavailable in some endemic areas and there are drug-resistant *Leishmania* strains (20).

There is an urgent need for new and safe components and drugs, therefore medical plants can be an alternative (20, 21). The use of plants for treating disease is as old as human history and frequently prescribed during the decades. The extracted components are used all around the world to treat health disorders and disease such as infectious forms (22). Plant extracts are widely used in Iran. Currently, about 820 herbal drugs are produced in Iran. Efficacy of some herbal plants like garlic, shallots, wormwood, yarrow, walnuts, thyme, henna plant, mimosa, aloe, wood betony and figwort have been shown on Iranian strain of *L. major* (MRHO/IR/75/ER) by In vitro and In vivo assessments (20, 23). However, the efficacy of some herbal plants has not been evaluated clinically and scientifically, but the interest for the use of plants, as safer and cheaper therapy exists (21).

*L. major* has been associated with zoonotic cutaneous leishmaniasis (ZCL) and it has high incidence rate in Iran (18). In present study, we evaluated in vitro anti-leishmanial effect of essential oils and nano-emulsions of *Lavander* and *Rosemary. Rosemary* extract has shown anti-tumor property as it can prevent cell proliferation. The researchers isolated carnosol and carnosic acid from *Rosemary* leaves which had anticancer property (24). In addition, *Rosemary* has antibacterial and anti-fungal effect and its anti-oxidant and anti-proliferative activity have been evaluated previously (24, 25). *Lavander* oils as antibacterial agents display good antibacterial activity against a range of bacteria including *Streptococcus, pyogenes*, *Enterobacter aerogenes*, *Staphylococcus aureus*, *Pseudomonas aeruginosa*, *Citrobacter freundii*, *Proteus vulgaris*, *Escherichia coli, VRE, Shigella sonnei* and *Propionibacterium acnes* (12). Our study revealed the sufficient effect of these components on *L. major* promastigote and amastigote stages.

This is the first time that essential oils and nano-emulsions of *Lavander* and *Rosemary* evaluated on *L. major* in Iran. We found different sensitivity of this parasite to the component assayed. Several concentrations of *Lavander* and *Rosemary* essential oils had sufficient effect on promastigote and amastigote stages of *L. major* in at least IC_50_= 0.11 μl/mL and IC_50_=0.26 μl/mL respectively. In addition, nano-emulsions of *Lavander* and *Rosemary* affected promastigote and amastigote stages of *L. major* in at least IC_50_=0.11 μl/mL and IC_50_=0.08 μl/mL respectively. Both essential oil and nano-emulsion of *Lavander* on at least IC_50_=0.11 μl/mL affected the promastigote stage of *L. major* although *Rosemary* nanoemulsion had lower IC_50_ and it had more inhibitory effect on *L. major*. The IC_50_ of *Rosemary* essential oil is 4 times less than *Lavander*. In addition, IC_50_ of *Lavander* essential oil is 0.32 times more than *Rosemary* nano-emulsion. Results were compared with MA with IC_50_=197mg/mL and revealed that essential oils and nano-emulsions of both *Lavander* and *Rosemary* are more effective than MA.

**Fig. 1:**
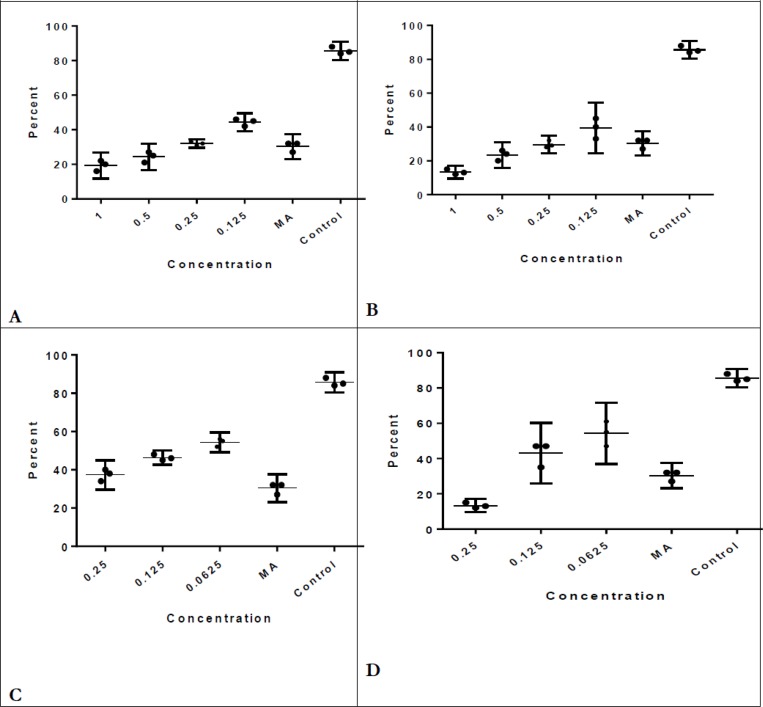
**A**) Several concentrations of Lavander essential oil and percent of infected macrophages (MIR) compared with MA and control. **B**) Several concentrations of Rosemary essential oil and percent of infected macrophages (MIR) compared with MA and control. **C**) Several concentrations of Lavander nano-emulsion and percent of infected macrophages (MIR) compared with MA and control. **D**) Several concentrations of Rosemary nano-emulsion and percent of infected macrophages (MIR) compared with MA and control. MA= meglumine antimoniate ; Control= infected macrophages without drug

In amastigote stage of parasite, *Lavander* and *Rosemary* essential oils were effective at least in concentration of 0.12 μl/mL and significantly reduced MIR (*P*=0.0001)(Fig. 1. A,B). In addition, *Lavander* and *Rosemary* nano-emulsion reduced MIR by at least 0.06 μl/mL which was significantly lower than control (*P*=0.0001). Obviously, the effective concentration of nano-emulsion was lower than essential oil in both components to reduce MIR (Fig. 1. C,D). Nano-emulsion of both *Lavander* and *Rosemary* has the same effect on decreasing the rate of infected macrophages but there is a little difference in concentration of 0.25μl/mL and *Rosemary* nano-emulsion was more effective than *Lavander* in this concentration (13.33% versus 37.33%).

**Fig. 2:**
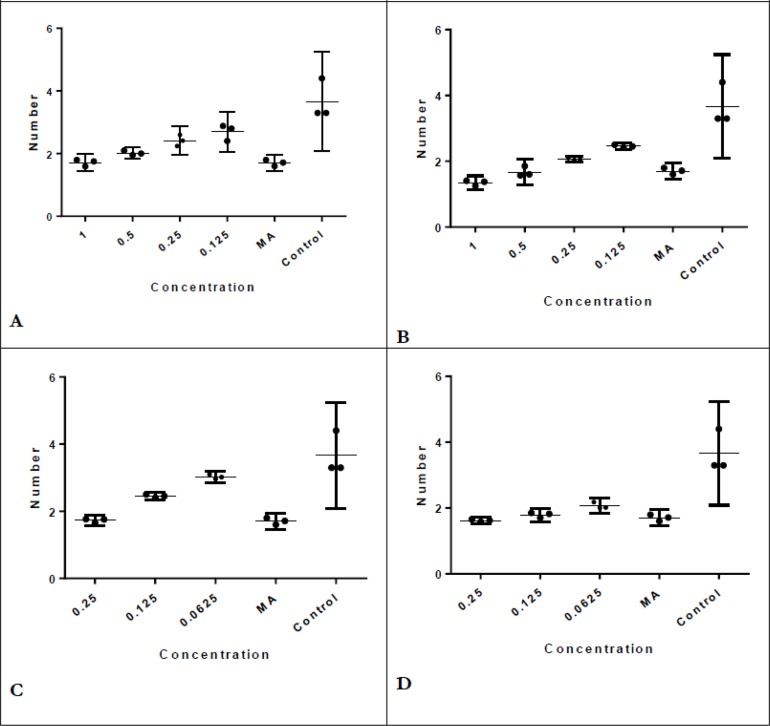
**A**) Several concentrations of Lavender essential oil and number of amastigotes in macrophages compared with MA and control. **B**) Several concentrations of Rosemary essential oil and number of amastigotes in macrophages compared with MA and control. **C**) Several concentrations of Rosemary nano-emulsion and number of amastigotes in macrophages compared with MA and control. **D**) Several concentrations of Lavander nano-emulsion and number of amastigotes in macrophages compared with MA and control. MA=meglumine antimoniate ; Control= infected macrophages without drug

*Lavander* essential oil significantly decreased the number of amastigotes in macrophages compared with control at least in 0.12 μl/mL, the difference between several concentrations and MA was not significant (*P*>0.05) (Fig. 2.A)(Table 2). Even in concentrations of 1μl/mL and 0.5 μl/mL of *Lavander* essential oil, MA decreased the number of amastigotes in macrophages significantly more. Results were achieved for *Rosemary* essential oil revealed in concentration 1μl/mL, the number of amastigotes in macrophages were significantly lower than MA (*P*=0.04) (Fig. 2. B) (Table 2).

*Lavander* and *Rosemary* nano-emulsions significantly decreased the number of amastigotes at least in 0.12 μl/mL (*P*=0.002) and 0.06 μl/mL (*P*=0.0001) in comparison with control (Fig. 2.C and D). *Rosemary* nanoemulsion is more effective than *Lavander* nanoemulsion in decreasing the number of amastigotes. After all, nano-emulsions were effective in lower concentrations than essential oils in both MIR and amastigotes in macrophages considered efficient components, but when we compared them with drug of choice (MA), *Rosemary* essential oil and nanoemulsion were more effective than *Lavander* in reducing MIR in concentration 0.25 μl/mL (29.6% versus 32%) (*P*=0.49)(Fig. 1. C). *Lavander* and *Rosemary* essential oils were compared and in concentrations 1 μl/ml, 0.5 μl/ml, 0.25 μl/ml *Rosemary* essential oil decreased the number of amastigotes in macrophages significantly more than *Lavander* (*P*=0.04).

Nanoemulsions of *Lavander* and *Rosemary* were compared and statistically significant difference observed as in concentrations 0.12 μl/mL and 0.06 μl/mL, the number of amastigotes in macrophages with *Rosemary* nano-emulsion was less than *Lavander* (*P*=0.04)(Fig. 2 C and D). There are limited studies carried out on *Lavander* and *Rosemary* extracts.

In a study carried out on atopic dermatitis in induced mice, the researchers evaluated the effect of *Lavander* and *thyme* essential oils on oxidative stress and immunity in disease. Their results showed the efficient effect of *Lavander* essential oil and the mixture with *thyme* in controlling disease symptoms (26). Another study with *Rosemary* extracts on young rats revealed that the extract had no effect on enhancing general immunity but may be effective in some conditions of stress like anti-oxidant or protein deficiency (27).

*Lavander* has antiparasitic effect as in primary investigation with two *Lavander* including *L. angustifolia* and *L.* x*intermedia* essential oils against three protozoal pathogens *Giardia duodenalis,* and *Trichomonas vaginalis* and the fish pathogen *Hexamita inflata*, the efficacy of *Lavander* was evaluated. In addition, *L. angustifolia* essential oil has activity against mite, grain weevils, aphids, and clothes moth. Research on the use of essential oils and other plant extracts on protozoan pathogens are limited to *Plasmodium* spp and *Leishmania* spp (12, 28).

According to our preliminary evaluation, essential oils and nano-emulsions of both *Lavander* and *Rosemary* decreased the number of infected macrophages and the number of amastigotes in macrophages compared with control, and in cases described earlier more than MA. In addition, *Rosemary* nano-emulsion was more effective than *Lavander*. Low concentration of nano-emulsions and essential oils compared with MA and amazing results showed the good efficacy of both *Rosemary* and *Lavander* nano-emulsions and essential oils on *L.major.* These formulations could be used with MA in combination therapy to reduce the time of healing.

## Conclusion

The nano-emulsions of both *L. angustifolia* and *R. officinalis* medicinal plants were more effective than essential oil in both MIR and amastigote. As a whole, clinical assessment of the anti-leishmanial activity of these plants on murine model is recommended.
